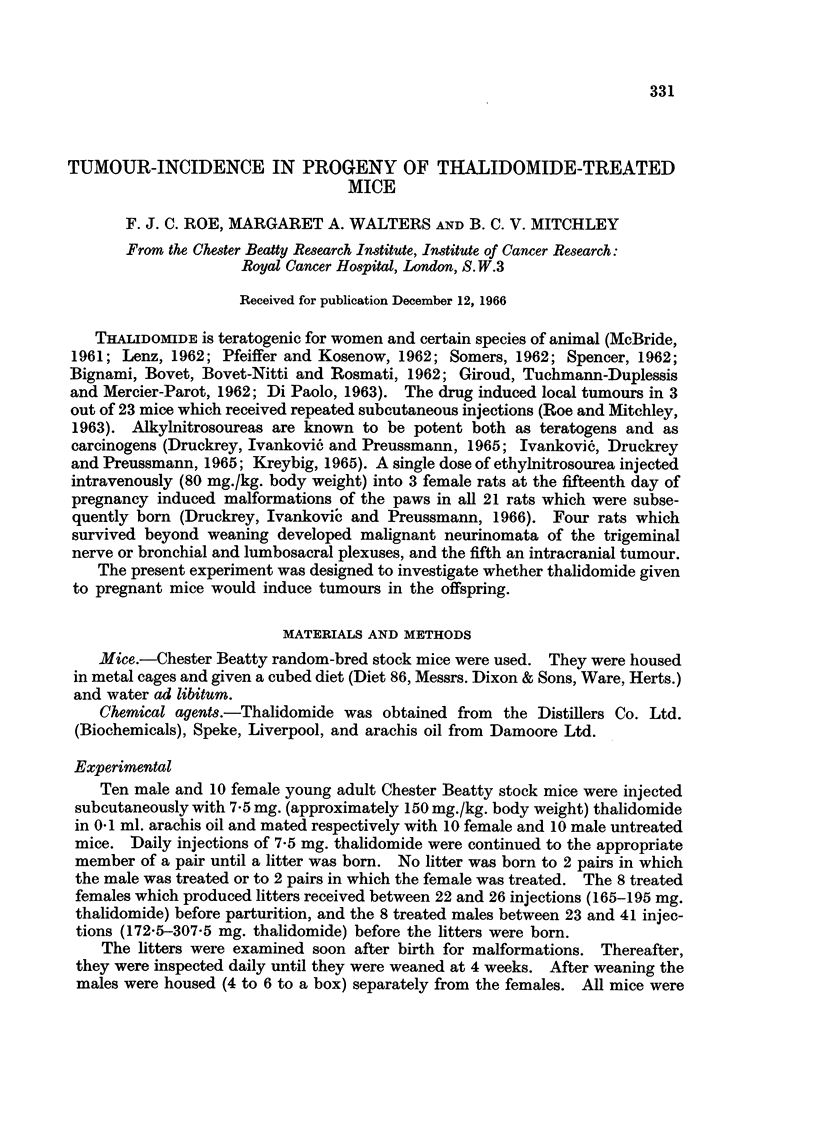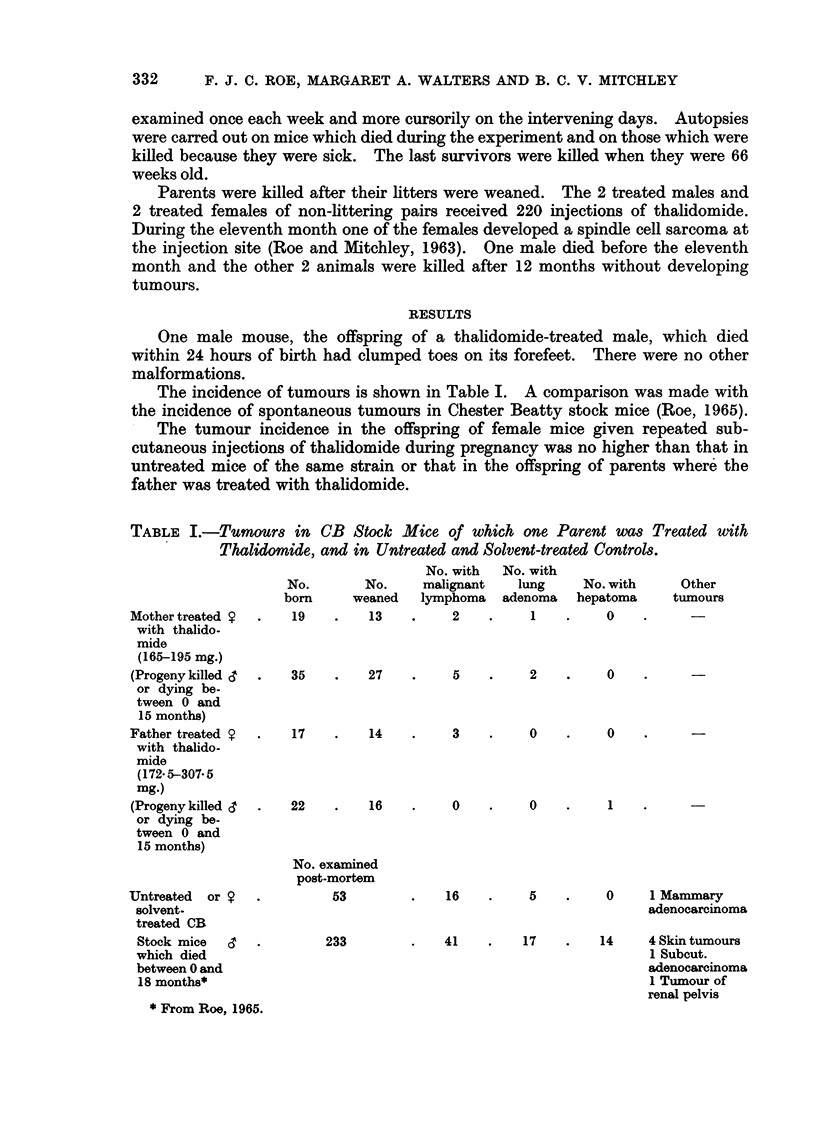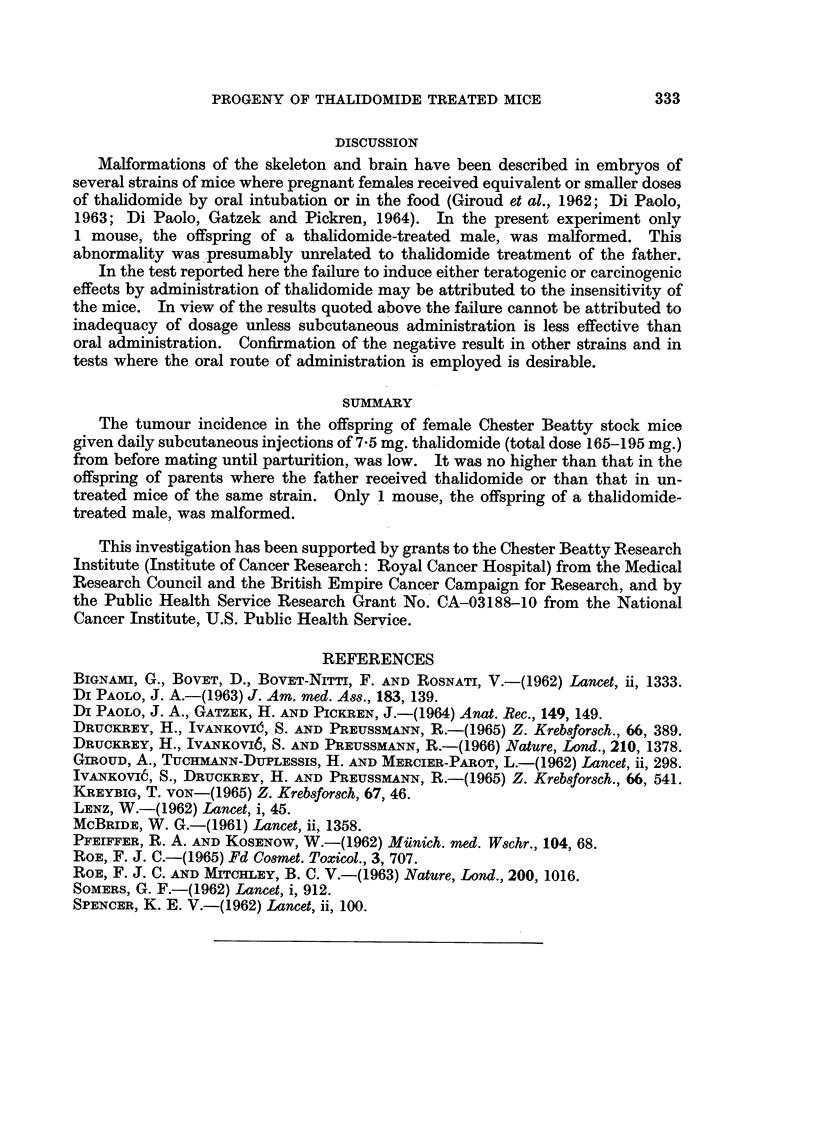# Tumour-incidence in progeny of thalidomide-treated mice.

**DOI:** 10.1038/bjc.1967.36

**Published:** 1967-06

**Authors:** F. J. Roe, M. A. Walters, B. C. Mitchley


### TUMOUR-INCIDENCE IN PROGENY OF THALIDOMIDE-TREATED MICE

F. J. C. ROE, MARGARET A. WALTERS AND B. C. V. MITCHLEY

*From the Chester Beatty Research Institute, Institute of Cancer Research: Royal Cancer Hospital, London, S.W.3*

Received for publication December 12, 1966

THALIDOMIDE is teratogenic for women and certain species of animal (McBride, 1961; Lenz, 1962; Pfeiffer and Kosenow, 1962; Somers, 1962; Spencer, 1962; Bignami, Bovet, Bovet-Nitti and Rosmati, 1962; Giroud, Tuchmann-Duplessis and Mercier-Parot, 1962; Di Paolo, 1963). The drug induced local tumours in 3 out of 23 mice which received repeated subcutaneous injections (Roe and Mitchley, 1963). Alkylnitrosoureas are known to be potent both as teratogens and as carcinogens (Druckrey, Ivanković and Preussmann, 1965; Ivanković, Druckrey and Preussmann, 1965; Kreybig, 1965). A single dose of ethylnitrosourea injected intravenously (80 mg./kg. body weight) into 3 female rats at the fifteenth day of pregnancy induced malformations of the paws in all 21 rats which were subsequently born (Druckrey, Ivanković and Preussmann, 1966). Four rats which survived beyond weaning developed malignant neurinomata of the trigeminal nerve or bronchial and lumbosacral plexuses, and the fifth an intracranial tumour.

The present experiment was designed to investigate whether thalidomide given to pregnant mice would induce tumours in the offspring.

### MATERIALS AND METHODS

*Mice.*—Chester Beatty random-bred stock mice were used. They were housed in metal cages and given a cubed diet (Diet 86, Messrs. Dixon & Sons, Ware, Herts.) and water *ad libitum.*

*Chemical agents.*—Thalidomide was obtained from the Distillers Co. Ltd. (Biochemicals), Speke, Liverpool, and arachis oil from Damoore Ltd.

### *Experimental*

Ten male and 10 female young adult Chester Beatty stock mice were injected subcutaneously with 7·5 mg. (approximately 150 mg./kg. body weight) thalidomide in 0·1 ml. arachis oil and mated respectively with 10 female and 10 male untreated mice. Daily injections of 7·5 mg. thalidomide were continued to the appropriate member of a pair until a litter was born. No litter was born to 2 pairs in which the male was treated or to 2 pairs in which the female was treated. The 8 treated females which produced litters received between 22 and 26 injections (165–195 mg. thalidomide) before parturition, and the 8 treated males between 23 and 41 injections (172·5–307·5 mg. thalidomide) before the litters were born.

The litters were examined soon after birth for malformations. Thereafter, they were inspected daily until they were weaned at 4 weeks. After weaning the males were housed (4 to 6 to a box) separately from the females. All mice were examined once each week and more cursorily on the intervening days. Autopsies were carred out on mice which died during the experiment and on those which were killed because they were sick. The last survivors were killed when they were 66 weeks old.

Parents were killed after their litters were weaned. The 2 treated males and 2 treated females of non-littering pairs received 220 injections of thalidomide. During the eleventh month one of the females developed a spindle cell sarcoma at the injection site (Roe and Mitchley, 1963). One male died before the eleventh month and the other 2 animals were killed after 12 months without developing tumours.

### RESULTS

One male mouse, the offspring of a thalidomide-treated male, which died within 24 hours of birth had clumped toes on its forefeet. There were no other malformations.

The incidence of tumours is shown in Table I. A comparison was made with the incidence of spontaneous tumours in Chester Beatty stock mice (Roe, 1965).

The tumour incidence in the offspring of female mice given repeated subcutaneous injections of thalidomide during pregnancy was no higher than that in untreated mice of the same strain or that in the offspring of parents where the father was treated with thalidomide.

TABLE I.—*Tumours in CB Stock Mice of which one Parent was Treated with Thalidomide, and in Untreated and Solvent-treated Controls.*

| | | No. born | No. weaned | No. with malignant lymphoma | No. with lung adenoma | No. with hepatoma | Other tumours |
|---|---|---|---|---|---|---|---|
| Mother treated with thalidomide (165–195 mg.) | ♀ | 19 | 13 | 2 | 1 | 0 | — |
| (Progeny killed or dying between 0 and 15 months) | ♂ | 35 | 27 | 5 | 2 | 0 | — |
| Father treated with thalidomide (172·5–307·5 mg.) | ♀ | 17 | 14 | 3 | 0 | 0 | — |
| (Progeny killed or dying between 0 and 15 months) | ♂ | 22 | 16 | 0 | 0 | 1 | — |
| | | No. examined post-mortem | | | | | |
| Untreated or solvent-treated CB | ♀ | 53 | | 16 | 5 | 0 | 1 Mammary adenocarcinoma |
| Stock mice which died between 0 and 18 months* | ♂ | 233 | | 41 | 17 | 14 | 4 Skin tumours<br>1 Subcut. adenocarcinoma<br>1 Tumour of renal pelvis |

* From Roe, 1965.

### DISCUSSION

Malformations of the skeleton and brain have been described in embryos of several strains of mice where pregnant females received equivalent or smaller doses of thalidomide by oral intubation or in the food (Giroud *et al.*, 1962; Di Paolo, 1963; Di Paolo, Gatzek and Pickren, 1964). In the present experiment only 1 mouse, the offspring of a thalidomide-treated male, was malformed. This abnormality was presumably unrelated to thalidomide treatment of the father.

In the test reported here the failure to induce either teratogenic or carcinogenic effects by administration of thalidomide may be attributed to the insensitivity of the mice. In view of the results quoted above the failure cannot be attributed to inadequacy of dosage unless subcutaneous administration is less effective than oral administration. Confirmation of the negative result in other strains and in tests where the oral route of administration is employed is desirable.

### SUMMARY

The tumour incidence in the offspring of female Chester Beatty stock mice given daily subcutaneous injections of 7·5 mg. thalidomide (total dose 165–195 mg.) from before mating until parturition, was low. It was no higher than that in the offspring of parents where the father received thalidomide or than that in untreated mice of the same strain. Only 1 mouse, the offspring of a thalidomide-treated male, was malformed.

This investigation has been supported by grants to the Chester Beatty Research Institute (Institute of Cancer Research: Royal Cancer Hospital) from the Medical Research Council and the British Empire Cancer Campaign for Research, and by the Public Health Service Research Grant No. CA–03188–10 from the National Cancer Institute, U.S. Public Health Service.

### REFERENCES

Bignami, G., Bovet, D., Bovet-Nitti, F. and Rosnati, V.—(1962) *Lancet*, ii, 1333.
Di Paolo, J. A.—(1963) *J. Am. med. Ass.*, **183**, 139.
Di Paolo, J. A., Gatzek, H. and Pickren, J.—(1964) *Anat. Rec.*, **149**, 149.
Druckrey, H., Ivanković, S. and Preussmann, R.—(1965) *Z. Krebsforsch.*, **66**, 389.
Druckrey, H., Ivanković, S. and Preussmann, R.—(1966) *Nature, Lond.*, **210**, 1378.
Giroud, A., Tuchmann-Duplessis, H. and Mercier-Parot, L.—(1962) *Lancet*, ii, 298.
Ivanković, S., Druckrey, H. and Preussmann, R.—(1965) *Z. Krebsforsch.*, **66**, 541.
Kreybig, T. von—(1965) *Z. Krebsforsch*, **67**, 46.
Lenz, W.—(1962) *Lancet*, i, 45.
McBride, W. G.—(1961) *Lancet*, ii, 1358.
Pfeiffer, R. A. and Kosenow, W.—(1962) *Münich. med. Wschr.*, **104**, 68.
Roe, F. J. C.—(1965) *Fd Cosmet. Toxicol.*, **3**, 707.
Roe, F. J. C. and Mitchley, B. C. V.—(1963) *Nature, Lond.*, **200**, 1016.
Somers, G. F.—(1962) *Lancet*, i, 912.
Spencer, K. E. V.—(1962) *Lancet*, ii, 100.